# The Progress of Ferroptosis of Immune Cells in the Tumor Microenvironment and Its Impact on Tumorigenesis and Development

**DOI:** 10.1002/iid3.70333

**Published:** 2026-01-20

**Authors:** Fenfen Zhan, Yanyan Hu, Xiang Jiang, Zejun Fang

**Affiliations:** ^1^ Endocrinology Department, Sanmen People's Hospital, Sanmenwan Branch, the First Affiliated Hospital Zhejiang University School of Medicine Sanmen China; ^2^ Clinical Laboratory Sanmen People's Hospital Sanmen China; ^3^ Department of Gastroenterology Sanmen People's Hospital Sanmen China; ^4^ Central Laboratory Sanmen People's Hospital Sanmen China

**Keywords:** cancer treatment, ferroptosis, immune cells, immunotherapy, therapeutic strategies, tumor immune microenvironment

## Abstract

**Background:**

The immune cells within the tumor microenvironment (TME) play important roles in tumorigenesis. Ferroptosis is an iron‐dependent form of non‐apoptotic cell death characterized by the accumulation of lipid peroxides. The interplay between ferroptosis and the tumor immune microenvironment significantly influences the outcome of cancer immunotherapy. The study aims to elucidate the dual effects of ferroptosis on cancer progression and immune responses, particularly in the context of enhancing the efficacy of tumor immunotherapy.

**Methods:**

An extensive literature review was conducted using PubMed to identify studies related to ferroptosis and immune cells in the TME, emphasizing translational research outcomes published within the last 5 years.

**Results:**

The study reviews the literature on the mechanisms of ferroptosis and its interactions with various components of the TME, including immune cells such as CD8+ T cells, dendritic cells, natural killer cells, regulatory T cells, myeloid‐derived suppressor cells, and tumor‐associated macrophages. It also examines the impact of ferroptosis inducers and inhibitors on these interactions, alongside the potential synergistic effects of combining ferroptosis induction with current immunotherapies. Ferroptosis plays a dual role in the TME by both promoting and inhibiting tumor growth through its effects on immune cell function. Activation of ferroptosis in tumor cells can enhance the immunogenicity of cancer cells, thereby improving the effectiveness of immunotherapies. Conversely, ferroptosis in immune cells can lead to immune cell dysfunction and contribute to immunotherapy resistance. The study identifies several therapeutic strategies that harness the induction of ferroptosis to overcome resistance to immune checkpoint inhibitors and enhance the anti‐tumor immune response. Inducing ferroptosis in tumor cells and immunosuppressive cells, while preventing ferroptosis in effector immune cells, emerges as a promising strategy to enhance the efficacy of immunotherapy.

**Conclusion:**

This review highlights the potential of targeting ferroptosis as a sensitization approach to improve cancer treatment outcomes, underscoring the need for further research to fully understand the regulatory mechanisms of ferroptosis in tumor immunity.

## Summary

- Highlights ferroptosis's dual role in promoting and inhibiting tumor growth through immune system interactions, affecting immunotherapy outcomes.
- Identifies ferroptosis induction as a promising strategy to overcome immunotherapy resistance, emphasizing its potential to enhance anti‐tumor responses.
- Examines ferroptosis's influence on crucial immune cells within the tumor microenvironment, showcasing its complex role in cancer progression and immune response.
- Suggests targeting ferroptosis as an innovative approach to sensitize cancer treatments, advocating for further research to exploit its full potential in tumor immunity.

## Graphical abstract

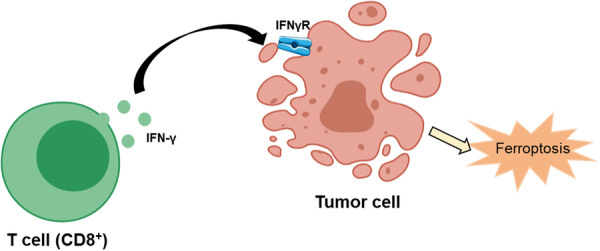

**Abbreviations**
ACSL4: acyl‐CoA synthetase long‐chain family member 4
ASAH2: N‐acylsphingosine amidohydrolase 2
ATP: adenosine triphosphate
CTLs: CD8+ cytotoxic T lymphocytes
DAMPs: damage‐associated molecular patterns
DCs: dendritic cells
FSP1: ferroptosis suppressor protein 1
GPX4: glutathione peroxidase 4
GSH: glutathione
HMGB1: high mobility group box 1
HMOX1: heme oxygenase 1
IFN‐γ: interferon‐gamma
iNOS: inducible nitric oxide synthase
MDSCs: myeloid‐derived suppressor cells
NK: natural killer
NO: nitric oxide
oxLDL: oxidized low‐density lipoprotein
P2X7: purinergic receptor P2X7
PD‐1: programmed death‐1
PD‐L1: programmed death‐ligand 1
SLC3A2: solute carrier family 3 member 2
SLC7A11: solute carrier family 7 member 11
TAMs: tumor‐associated macrophages
TLR4: Toll‐Like Receptor 4
Tregs: regulatory T cells
TYRO3: TYRO3 protein tyrosine kinase
XCT: cystine/glutamate reverse transporter

## 1 Introduction

Ferroptosis represents a novel form of cell death characterized by the excessive accumulation of ferrous ions and lipid peroxides [1]. It encompasses multiple cellular processes, including the progression of cancer. The induction of ferroptosis in tumor cells is regarded as a new approach for eradicating tumor cells, especially those resistant to radiotherapy and chemotherapy [2]. Therefore, a comprehensive exploration of the role and impact of ferroptosis in cancer, as well as the mechanisms underlying the application of ferroptosis in cancer therapy, holds significant importance for identifying effective therapeutic targets and developing novel treatment strategies [3].

The tumor microenvironment, consisting of the cellular milieu surrounding tumor cells such as endothelial cells, immune cells, fibroblasts, mesenchymal stem cells, and the extracellular matrix, plays a critical role [4]. Particularly, the immune microenvironment, shaped by immune cells and their secreted factors, is key to tumor proliferation and metastasis, with tumor immune evasion allowing escape from immune cell attack [4]. Currently, several conventional clinical drugs, such as the liver cancer‐targeting drug sorafenib and the anti‐inflammatory drug for inflammatory bowel disease, sulfasalazine, promote tumor cell ferroptosis by targeting key signaling pathways like Glutathione Peroxidase 4 (GPX4). Experimental drugs, including small‐molecule inhibitors like RAS‐selective lethal molecule 3 and DHODH, enhance ferroptosis by targeting the ferroptosis defense system [5, 6]. Recent advancements in tumor immunotherapy, particularly immune checkpoint inhibitors and adoptive cell therapy, have significantly improved the survival rates of cancer patients [2, 7]. However, only a small subset of patients responds to immunotherapy, attributed to low immunogenicity of tumor cells and the presence of an immunosuppressive microenvironment, which limits the anti‐tumor effects of immunotherapy [5, 8]. The tumor microenvironment, a complex and dynamic landscape primarily composed of tumor cells and infiltrating immune cells, influences the effectiveness of immunotherapy [9]. Increased tumor‐associated macrophages (TAMs), myeloid‐derived suppressor cells (MDSCs), regulatory T cells, and decreased cytotoxic T cells contribute to tolerance or non‐responsiveness to immune therapy [10].

Given the intricate links between ferroptosis and tumor‐infiltrating immune cells, this article primarily elaborates on the role of ferroptosis within the tumor immune microenvironment, providing a theoretical basis for the feasibility of combining ferroptosis therapy with tumor immunotherapy. Ferroptosis describes an iron‐dependent process of regulated cell death characterized by the excessive accumulation of lipid reactive oxygen species [11]. Dolma et al. [12] discovered a novel compound, erastin, which selectively induces death in tumor cells expressing the Ras gene, distinct from traditional cell death mechanisms due to the absence of nuclear morphology changes, DNA fragmentation, and caspase activation. This cell death process is not reversible by caspase inhibitors. Subsequent studies revealed that this form of cell death could be inhibited by iron chelators and identified another compound, RSL3, capable of inducing similar cell death [13]. In 2012, based on the mechanism by which erastin induces cell death in tumor cells with Ras mutations, this mode of cell death was officially named ferroptosis [14]. Erastin induces ferroptosis by inhibiting cellular cystine uptake, increasing the consumption of intracellular glutathione (GSH), and inactivating glutathione peroxidase 4 (GPX4), leading to the accumulation of lipid peroxidation and cell death [15]. Morphologically, ferroptosis is characterized by the integrity of the nuclear structure, absence of cell membrane swelling and rupture, along with mitochondrial shrinkage, increased membrane density, and diminished or absent mitochondrial cristae, distinguishing it from apoptosis, autophagy, and necrosis [16]. Studies have shown that the sensitivity of cells to ferroptosis is related to various biological pathways, including amino acid and glutathione metabolism, iron metabolism, and lipid metabolism [17]. There has been an exponential growth in the number of published studies on ferroptosis in cancer cells. The interplay between ferroptosis and the tumor immune microenvironment significantly influences the outcome of cancer immunotherapy. Inducing ferroptosis in tumor cells and immunosuppressive cells, while preventing ferroptosis in effector immune cells, emerges as a promising strategy to enhance the efficacy of immunotherapy. This review highlights the potential of targeting ferroptosis as a sensitization approach to improve cancer treatment outcomes, underscoring the need for further research to fully understand the regulatory mechanisms of ferroptosis in tumor immunity.

## 2 Principal Immune Cells in the Tumor Immune Microenvironment

The tumor immune microenvironment encompasses not only tumor cells but also immune cells, tumor‐associated fibroblasts, signaling molecules, and the extracellular matrix [18]. The immune cells mainly include CD8+ cytotoxic T lymphocytes (CTLs), dendritic cells (DCs), natural killer (NK) cells, regulatory T cells (Tregs), MDSCs, and TAMs. NK cells play a crucial role in the early stages of tumor eradication, serving as the body's first line of defense against tumors by recognizing non‐specific antigens on the surface of tumor cells through their activating or inhibitory receptors and directly killing these cells [19]. Furthermore, CD8+ T cells, with the assistance of antigen‐presenting cells, are activated and differentiate into CTLs. CTLs are the primary immune cells involved in killing tumor cells, doing so by secreting cytotoxic molecules such as perforin and granzymes. Besides the anti‐tumor immune cells, the body also harbors suppressive immune cells. Tregs are among the most extensively studied suppressive immune cells. They inhibit the proliferation and function of target immune cells and promote tumor immune evasion by secreting negative cytokines such as interleukin‐10 (IL‐10) and IL‐4, or by directly interacting with target immune cells through CTLA‐4 and transforming growth factor‐beta (TGF‐β) [20]. Similar to Tregs, MDSCs also exert immunosuppressive effects by inhibiting CTLs and NK cell functions through the secretion of arginase, reactive oxygen species, and nitric oxide synthase. TAMs can be categorized into M1 and M2 types [21]. M1 macrophages, which are more prevalent in early‐stage tumors, exhibit anti‐tumor activity. In the tumor microenvironment, the role of ferroptosis may vary depending on cell types and different tumor contexts. Ferroptosis is believed to have pro‐tumor effects in some cases, while in others, it may suppress tumor growth. The current role of ferroptosis in tumors is a double‐edged sword. How to rationally utilize the mechanism of ferroptosis to prevent and treat cancer requires systematic analysis and exploration. Therefore, further clarification of ferroptosis's dual role in various microenvironments is crucial for a deeper understanding of its potential in cancer treatment. However, as the tumor progresses, M1 macrophages in the tumor microenvironment gradually convert to M2 macrophages. Research has confirmed that M2‐type TAMs promote tumor angiogenesis and play a role in facilitating tumor progression. And, it's crucial to highlight that the tumor immune microenvironment not only orchestrates the battle between tumor‐promoting and tumor‐suppressing forces but also significantly influences ferroptosis, a form of regulated cell death [22, 23]. The interactions between immune cells and the mechanisms of ferroptosis are integral to understanding tumor progression and devising novel therapeutic strategies.

## 3 Ferroptosis Exerts a Dual Role in the Tumor Immune Microenvironment

Ferroptosis plays a dual role in the tumor immune microenvironment, characterized by increased unstable iron, lipid peroxidation, aberrant accumulation of reactive oxygen species, and redox system imbalance in cancer cells, all of which can accelerate the ferroptosis process [24]. The metabolites produced by ferroptosis interact with various effector immune cells and immune molecules within the tumor immune microenvironment, exhibiting a dual role in tumor immunity [25]. On the one hand, research shows that immunotherapy inhibits tumor growth by inducing ferroptosis in tumor cells. For example, immunotherapy can enhance the level of specific lipid peroxidation in tumor cells by activating CD8+ T cells, thereby inducing ferroptosis in tumor cells [26]. PD‐L1 blockade can promote lipid peroxidation‐dependent ferroptosis in cancer cells; PD‐L1 antibodies and ferroptosis inducers synergistically inhibit tumor growth in vitro and in vivo [27]. Knockdown of TYRO3 promotes ferroptosis in tumor cells and makes drug‐resistant tumors sensitive to PD‐1 treatment, which can overcome anti‐PD‐1/PD‐L1 resistance [28]. Currently, most studies focus on the therapeutic role of ferroptosis in tumor treatment.

On the other hand, ferroptosis may have a cancer‐promoting effect under certain circumstances. For instance, in pancreatic ductal adenocarcinoma, ferroptosis leads to the release of KRASG12D into exosomes, which can be taken up by macrophages, promoting the polarization and activation of M2‐type macrophages through activation of the STAT3 pathway, thereby facilitating tumor growth and limiting antitumor immunity [29]. Damage‐associated molecular patterns (DAMPs) are endogenous cellular molecules that participate in various physiological processes under normal conditions. Upon tumor cell damage or death, DAMPs are released and acquire immunogenicity, capable of binding to pattern recognition receptors such as Toll‐like receptor 4 (TLR4) and the purinergic receptor P2X7, promoting inflammation and activation of the innate immune system. Originating from cell membranes and mitochondria, DAMPs are also products of the extracellular matrix [30]. Common DAMPs like adenosine triphosphate (ATP), high mobility group box 1 (HMGB1), and calreticulin play a significant role as adjuvants in immunogenic cell death. These molecules bind to receptors such as P2X7, TLR4, or LDL receptor‐related protein 1, thereby activating the immune system and enhancing anti‐tumor immune responses [31]. Besides stimulating tumor immunity, some DAMPs may also promote tumor growth.

## 4 The Effect of Ferroptosis on Immune Cell Function and Phenotype

### 4.1 The Immunoregulatory Role of Ferroptosis in TAMs

**Figure 1:**
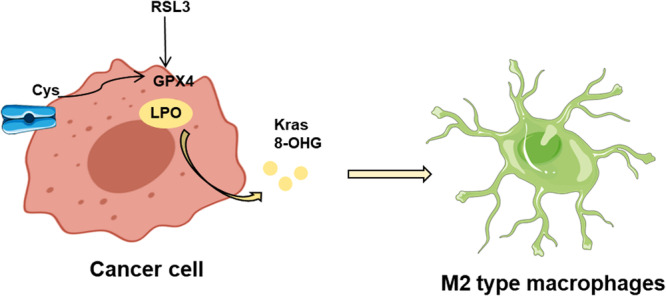
Ferroptosis regulates M2 macrophage polarization.

TAMs are primarily categorized into anti‐tumoral M1 macrophages and pro‐tumoral M2 macrophages. Macrophages within the tumor microenvironment can be broadly classified into two main subtypes: M1 macrophages, which are pro‐inflammatory and exhibit anti‐tumor activity, and M2 macrophages, which are anti‐inflammatory and promote tumor progression [32]. M1 macrophages are characterized by high levels of inducible nitric oxide synthase (iNOS) and the production of pro‐inflammatory cytokines, while M2 macrophages express markers such as CD206 and produce anti‐inflammatory cytokines like IL‐10 and TGF‐β [33]. Previous research has confirmed that M1 and M2 macrophages exhibit different sensitivities to ferroptosis. Compared to M2 macrophages, M1 macrophages express higher levels of iNOS and produce more nitric oxide (NO) radicals [34, 35]. NO radicals can react with lipid radicals, reducing intracellular lipid peroxidation and inhibiting ferroptosis. In pancreatic cancer, the proportion of M2 macrophages is significantly higher than that of M1 macrophages. Studies have found that tumor cells can secrete the KRASG12D protein in the form of exosomes into the extracellular environment [36]. This protein can be ingested by macrophages and promotes fatty acid peroxidation via a STAT3‐mediated mechanism inside the cell, inducing macrophage differentiation into the M2 type and facilitating tumor immune evasion [37]. Given the anti‐tumoral functions and proportions of M1 macrophages, an increasing number of researchers are investigating how to convert M2 macrophages into M1 macrophages [38]. It has been discovered that the use of iron‐containing organic framework nanoparticles and ferroptosis inducers, or zero‐valent iron nanoparticles, can induce the conversion of M2 macrophages to M1 macrophages. Research indicates that macrophages exhibit different sensitivities to ferroptosis inducers. M1‐like macrophages with high oxidative stress (OS) expressions are more tolerant to ferroptosis; meanwhile, M2 macrophages are relatively more sensitive to ferroptosis [26, 39]. The figure depicts how ferroptosis in cancer cells can lead to the polarization of macrophages toward the M2 subtype, facilitated by the release of Kras and 8‐OHG, thus contributing to tumor progression (Figure 1) [40]. In addition, different macrophage subtypes exhibit varying tolerances to ferroptosis due to differences in energy metabolism; for instance, M1 macrophages avoid ferroptosis due to high expression of iNOS. Thus, by reprogramming the energy metabolism of macrophages, it is possible to induce ferroptosis in M2 macrophages or promote polarization and activation of M1 macrophages, thereby enhancing the efficacy of tumor immunotherapy [41].

### 4.2 Research on Ferroptosis in MDSCs

MDSCs originate from bone marrow progenitor cells and immature myeloid cells, serving as precursors to monocytes, DCs, and granulocytes [42]. In cancer patients, tumor‐derived inflammatory factors activate MDSCs, which then exert immunosuppressive effects, facilitating tumor immune evasion. Previous research has indicated that N‐acylsphingosine amidohydrolase (ASAH2) is highly expressed in MDSCs. By negatively regulating the level of the p53 protein and inhibiting the expression of HMOX1 protein, ASAH2 ultimately reduces the generation of reactive oxygen species, preventing ferroptosis in MDSCs. In addition, MDSCs monopolize the interstitial cysteine by absorbing it, thereby inhibiting antigen‐presenting cells from secreting cysteine into the interstitium. These mechanisms lead to T cells being unable to acquire sufficient cysteine from the interstitium, significantly inhibiting T cell function [43].

### 4.3 Research on Ferroptosis in DCs

**Figure 2:**
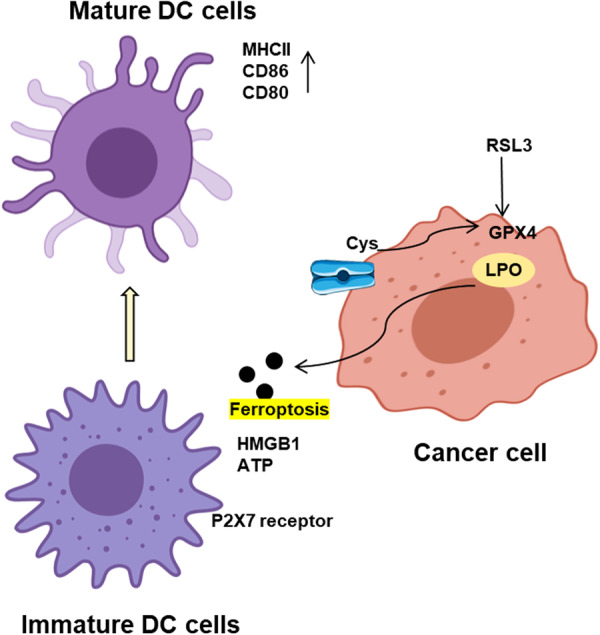
Ferroptosis regulates the differentiation and maturation of DCs.

DCs ingest and process tumor‐associated antigens, presenting them as MHC‐tumor antigen complexes to activate and promote T lymphocyte‐mediated tumor killing. DCs can also enhance the body's anti‐tumor immune response by promoting the proliferation of NK cells [44]. Previous studies suggest that tumor‐infiltrating DCs contain more lipids, making them more susceptible to ferroptosis, which in turn suppresses their antigen‐presenting function [45]. In addition, treatment with the GPX4 inhibitor RSL3 can induce DCs dysfunction, while knockout of PPARG can reverse this dysfunction and maintain their anti‐tumor effect [46]. In another study, Sestrin2 was shown to inhibit LPS‐induced DCs ferroptosis, thereby promoting DCs survival and function [47]. Early ferroptosis in tumor cells can enhance DC maturation and activation, improving their antigen‐presenting capability, and subsequently stimulating effector T cells to exert anti‐tumor effects. The GPX4 inhibitor RSI3 triggers immunogenic cell death (ICD) in tumor cells to activate DCs, introducing a new mechanism for inducing anti‐tumor immunity [48]. Pharmacological blocking of ATP receptors can reverse the anti‐tumor protective effect of early ferroptotic cancer cells, suggesting ATP is essential for ICD induced by early ferroptotic cancer cells [49]. The role of DCs in the tumor immune microenvironment is pivotal, as they serve as key antigen‐presenting cells that bridge innate and adaptive immunity. The study aims to explore how ferroptosis affects the functionality of DCs, particularly their antigen‐presenting capabilities, which are crucial for initiating effective anti‐tumor immune responses [50, 51]. The study hypothesizes that modulating ferroptosis in DCs could either impair or enhance their ability to stimulate T cells and NK cells, thereby influencing the overall anti‐tumor immune response [6, 52]. It shows the role of ferroptosis in the maturation of DCs, with immature DCs being converted into mature DCs characterized by increased expression of MHCII, CD86, and CD80 [53, 54]. The cancer cell undergoing ferroptosis, induced by agents like RSL3 through the inhibition of GPX4, releases DAMPs such as HMGB1 and ATP, which engage P2X7 receptors on DCs, further promoting their maturation (Figure 2) [55, 56].

### 4.4 The Role of Ferroptosis in NK Cells

NK cells, as innate immune cells, play a significant role in anti‐tumor immunity through mechanisms such as the perforin/granzyme pathway, the Fas‐FasL and TNF‐α‐TNFR‐1 pathways, and antibody‐dependent cell‐mediated cytotoxicity. Studies have found that tumor‐infiltrating NK cells contain higher levels of proteins related to ferroptosis and lipid oxidation [57]. Consequently, tumor‐infiltrating NK cells exhibit significantly increased oxidative stress and markedly reduced glucose metabolism, leading to NK cell dysfunction [58]. However, activating the NRF2 protein function in NK cells can reverse the suppressive effects of the tumor microenvironment on NK cells, reactivating their anti‐tumor functions [59].

Tumor cell ferroptosis can lead to the release of intracellular DAMPs, playing a dual role in anti‐tumor immunity. On one hand, DAMPs induce immunogenic cell death (ICD), enhancing the anti‐tumor functions of DCs and CD8+ T cells, while CD8+ T cells can in turn promote tumor cell ferroptosis by secreting IFN‐γ [60]. On the other hand, tumor cells release specific DAMPs and secrete exosomes containing high levels of PD‐1, stimulating TAMs to transform into an M2‐like anti‐inflammatory, tumor‐promoting phenotype, thereby facilitating tumor development and progression [52]. Besides tumor cells undergoing ferroptosis to affect immune responses, immune cells in the microenvironment can also undergo ferroptosis, contributing to resistance to immune therapy. For example, exhausted T cells with high expression of CD36 can lead to an increase in intracellular oxidized lipids, causing T cell ferroptosis and resistance to immune therapy [25]. The study highlights that activating the NRF2 pathway in NK cells can counteract the suppressive effects of the tumor microenvironment, thereby restoring their anti‐tumor functions [61, 62]. This suggests that NRF2 activation or other strategies to reduce oxidative stress could be explored as therapeutic approaches to maintain or enhance NK cell activity in cancer patients. Furthermore, the dual role of ferroptosis in modulating immune responses—both through the release of DAMPs that promote immunogenic cell death (ICD) and through the potential induction of immune cell dysfunction—illustrates the complex interplay between ferroptosis and tumor immunity [63]. By understanding these mechanisms, the study provides insights into how ferroptosis can either be harnessed to boost anti‐tumor immunity or mitigated to prevent immune cell dysfunction, offering new avenues for improving the efficacy of immunotherapies targeting NK cells and other components of the immune system [64].

### 4.5 The Immunoregulatory Role of Ferroptosis in Tregs

Regulatory T cells (Tregs) are a subset of T cells that control the body's autoimmunity, primarily comprising thymus‐derived natural Tregs and peripherally induced Tregs [38]. Tregs play a crucial role in maintaining immune tolerance within the body. However, within the tumor microenvironment, Tregs promote tumor immune evasion by suppressing the body's immune response through the secretion of cytokines such as IL‐10 and TGF‐β. Research has shown that GPX4 is essential for maintaining the immunosuppressive function of Tregs. Knocking out GPX4 in Tregs does not affect the survival of Treg cells but increases the levels of lipid oxidation and ferroptosis within the cells, inhibiting the immunoregulatory function of Tregs. Treg cells with GPX4 knocked out enhance the TH17 cell response by secreting IL‐1β, promoting the body's anti‐tumor immune function [39]. The observed increase in lipid oxidation and ferroptosis upon GPX4 knockout in Tregs is consistent with this hypothesis. By inducing ferroptosis, the study effectively diminished the immunosuppressive capabilities of Tregs, which are known to secrete IL‐10 and TGF‐β to suppress the immune response [65, 66]. Interestingly, the knockdown of GPX4 did not affect the survival of Tregs but specifically targeted their function, suggesting that ferroptosis selectively impairs the immunoregulatory role of Tregs without inducing widespread cell death. This functional impairment of Tregs was further linked to an enhanced TH17 cell response, marked by increased secretion of IL‐1β, which aligns with the study's hypothesis that reducing Treg‐mediated suppression could invigorate anti‐tumor immunity [55, 67]. These findings support the broader hypothesis that ferroptosis induction within immunosuppressive cells, such as Tregs, could be a viable strategy to overcome immune evasion in the tumor microenvironment. By disrupting the regulatory functions of Tregs, ferroptosis shifts the immune balance toward an anti‐tumor response, potentially improving the efficacy of existing cancer immunotherapies [68, 69].

### 4.6 The Immunoregulatory Role of Ferroptosis in CTLs

A study found that immune checkpoint inhibitors activate and induce CD8+ T cells to secrete interferon‐gamma (IFN‐γ), which negatively regulates the expression of the Xc system components SLC3A2 and SLC7A11 on the surface of tumor cells. This action reduces the intracellular transport of cystine and the synthesis of intracellular glutathione, intensifies the level of intracellular lipid peroxidation, and promotes ferroptosis in tumor cells [59]. Artificially reducing the levels of cystine and cysteine in tumor cells can synergistically enhance the anti‐tumor effect of T cells. Another study suggests that ferroptosis inducers can enhance the anti‐tumor effect of radiotherapy. Overexpressing GPX4 or FSP1 enhances the resistance of CD8+ T cells to ferroptosis, thereby exerting their anti‐tumor immune function. Conversely, knocking out GPX4 in T cells exacerbates the lipid oxidation process within the cells, inhibiting their proliferative ability and immune function. These studies confirm that ferroptosis is involved in regulating the activity and function of CTLs, and CTLs influence the level of ferroptosis in tumor cells [57]. In the studies examining the immunoregulatory role of ferroptosis in CTLs, researchers utilized a combination of in vitro and in vivo experiments. In vitro studies often involved the use of cultured tumor cell lines and CTLs, where ferroptosis was induced using specific ferroptosis inducers such as erastin or RSL3 [26, 70, 71]. The expression levels of key proteins related to ferroptosis, such as GPX4 and SLC7A11, were manipulated through gene overexpression or knockdown techniques. Flow cytometry and Western blotting were used to assess changes in protein expression and cell viability [30, 72, 73]. In vivo studies typically employed mouse tumor models treated with immune checkpoint inhibitors and ferroptosis inducers, allowing the evaluation of tumor growth, CTLs function, and the interplay between ferroptosis and immune responses in the tumor microenvironment [74, 75]. These methods provided insights into the dual role of ferroptosis in modulating CTLs activity and its implications for enhancing tumor immunotherapy [76, 77]. However, there is debate over the sensitivity of tumor cells and CTLs to ferroptosis within the tumor microenvironment. Some studies believe that ferroptosis inducers can induce ferroptosis in tumor cells but have a minor effect on CTLs, suggesting that ferroptosis inducers can sensitize tumor immunotherapy. Yet, other research finds that GPX4 inhibitors can selectively induce ferroptosis in CTLs in in vitro models, thereby promoting tumor cell survival. This issue requires further research and exploration [57].

### 4.7 Interactions Between CD8+ T Cells and Ferroptosis

#### 4.7.1 CD8+ T Cells Facilitate Ferroptosis in Tumor Cells

**Figure 3:**
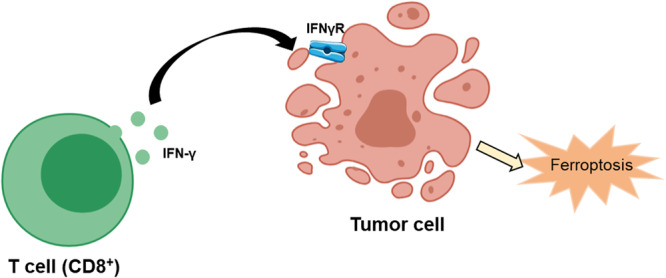
The mechanism of T cells inducing tumor cells' ferroptosis.

XCT, identified as a cystine/glutamate reverse transporter, primarily facilitates the intracellular import of cystine while exporting glutamate, participating in the biosynthesis of glutathione (GSH) and thereby influencing ferroptosis [78]. Studies indicate that CD8+ T cells activated by immunotherapy can downregulate the expression of XCT subunits SLC7A11 and SLC3A2 on tumor cell membranes through the secretion of interferon‐gamma (IFN‐γ), enhancing intracellular lipid peroxidation and leading to tumor cell ferroptosis [78]. This process results in the release of damage‐associated molecular patterns (DAMPs), further promoting the infiltration of CD8+ T cells within the tumor, thereby enhancing the anti‐tumor efficacy of immunotherapy [53]. Comparative analyses of the transcriptomes of patients treated with the PD‐1 inhibitor nivolumab before and during therapy revealed a decrease in SLC3A2 expression and an increase in IFN‐γ and CD8 expression, correlating with improved clinical outcomes [74]. CD8 + T cells‐derived IFN‐γ can induce cancer cells ferroptosis by binding to the surface IFN‐ γ receptor (IFNγR) and activating multiple pathways (Figure 3) [71]. This underscores the role of CD8+ T cells in promoting tumor cell ferroptosis and suggests that targeting the XCT/GSH/GPX4 pathway in combination with anti‐PD‐1/PD‐L1 antibodies may represent an effective tumor immunotherapy strategy [19].

Research indicates that the inhibition of tumor cell ferroptosis is also a mechanism of resistance to PD‐1/PD‐L1 inhibitors. The TCGA breast cancer cohort study corroborated that the expression of the ferroptosis‐related protein SLC3A2 is positively correlated with TYRO3 expression, and high TYRO3 expression is associated with resistance to PD‐1 inhibitors in breast cancer patients [36]. A recent study unveiled a novel mechanism by which CD8+ T cells kill tumor cells, proposing that arachidonic acid and IFN‐γ secreted by CD8+ T cells enhance the expression of acyl‐CoA synthetase long‐chain family member 4 (ACSL4), thereby promoting tumor cell ferroptosis and improving anti‐tumor immune efficacy [18]. The ACSL4 knockout mouse model demonstrated that, compared to the ACSL4 wild‐type, the ACSL4 knockout group exhibited reduced infiltration of CD8+ T cells within tumors, decreased expression of IFN‐γ and tumor necrosis factor alpha, lowered levels of intratumoral lipid peroxides, and significantly increased tumor volume [79]. This research not only provides new insights into the cytotoxic mechanisms of immune cells but also offers a novel combined strategy for inducing tumor cell ferroptosis and enhancing anti‐tumor immune efficacy.

#### 4.7.2 Ferroptosis Contributes to CD8+ T Cell Exhaustion

**Figure 4:**
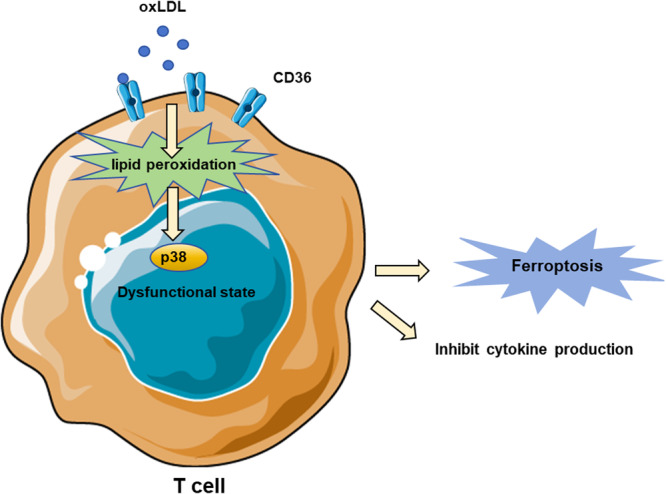
CD36 promoted uptake of oxLDL into T cells, and this induced lipid peroxidation and downstream activation of p38 kinase.

Ferroptosis is not only observed in tumor cells but also potentially occurs in infiltrating T cells within the immunosuppressive tumor microenvironment. The exhaustion of CD8+ T cells is associated with increased lipid uptake and the accumulation of intracellular lipid peroxides [80]. The research team led by Cui Guoliang recently identified a new tumor immunosuppression mechanism. The tumor microenvironment generates an abundance of oxidized low‐density lipoproteins (ox‐LDL), which are taken up by CD8+ T cells expressing high levels of CD36. This uptake increases intracellular lipid peroxidation, activating the stress‐response protein p38 and inducing T cell ferroptosis (Figure 4) [81]. Inhibition of CD36 or suppression of ferroptosis in CD8+ T cells significantly reinstated their antitumor functionality and, notably, demonstrated enhanced antitumor efficacy when combined with anti‐PD‐1 antibody therapy [82]. As a result, the expression of IFN‐β and TNF‐β is reduced, leading to CD8+ T cell dysfunction and a weakened anti‐tumor effect [71].

In addition, the research team discovered a positive correlation between CD86 expression and the immune checkpoint PD‐1/TIM‐3 expression [71], while overexpression of GPX4 could inhibit lipid peroxidation induced by ox‐LDL, rescuing T cells from ferroptosis and restoring their anti‐tumor function [83].

## 5 Ferroptosis and Immunotherapy

The induction of tumor cell ferroptosis can enhance anti‐tumor responses within the tumor microenvironment, making therapeutic strategies that combine ferroptosis induction with immunotherapy promising for cancer treatment [24, 25, 74]. Previous research has shown that in mice treated with PD‐L1 inhibitors alone, tumor growth is relatively reduced, and ferroptosis inducer sensitized tumors to PD‐L1 inhibitors treatment [84, 85]. Combination therapy significantly increases lipid peroxidation in tumor cells, elevates the levels of IFN‐γ, TNF‐β, CD8+ T, and CD4+ T cells, and enhances the anti‐tumor action of immune cells. However, recent studies have reported that during melanoma treatment with PD‐1 inhibitors, although tumor cell Xc function is inhibited, the secretion of exosomes with high levels of PD‐1 can stimulate M2 macrophage polarization, leading to resistance to PD‐1 inhibitors [42, 86]. Therefore, inhibiting the production of high levels of PD‐1‐containing exosomes in tumor cells is key to overcoming this resistance [87]. Using a nanocarrier system to deliver ferroptosis inducers and exosome inhibitors into the tumor microenvironment to promote tumor cell ferroptosis and restore T cell function represents a new therapeutic strategy to enhance tumor immunotherapy [36]. Constructing a nanocarrier that couples CW4869 exosome inhibitors with Fe^3+^ ferroptosis inducers, targeting B16F10 melanoma cells to inhibit exosome secretion, can indirectly suppress PD‐L1 expression, restore CD8+ T cell function, and promote IFN‐γ secretion. IFN‐β can reduce the expression of SLC7A11 and SLC3A2 on the tumor cell membrane, disrupt the tumor cell's antioxidant system, and induce tumor cell ferroptosis [71, 88].

### 5.1 Amino Acid and Glutathione Metabolism

GSH, an essential antioxidant in mammals, is synthesized from glutamate, glycine, and cysteine through the actions of glutamate‐cysteine ligase and glutathione synthetase. The depletion of GSH can trigger ferroptosis, which can be achieved by inhibiting the biosynthesis of GSH and blocking the intake of extracellular cystine (through the inhibition of system Xc‐ by erastin). The cystine‐glutamate antiporter system Xc is composed of the transmembrane proteins SLC3A2 and SLC7A11, facilitating the 1:1 exchange of extracellular cystine for intracellular glutamate, thereby reducing cystine to cysteine for GSH synthesis. Thus, inhibiting system Xc can trigger ferroptosis [89]. p53, one of the most extensively studied tumor suppressor genes, positively regulates ferroptosis by transcriptionally repressing system Xc [90]. In some cases, however, p53 can inhibit ferroptosis through a transcription‐independent mechanism by blocking the activity of the T‐cell surface antigen CD26, necessitating context‐specific assessment of p53's role. Another source of cysteine is through the reverse transsulfuration of methionine, enabling some cells to resist system Xc‐related ferroptosis [91].

### 5.2 Iron Metabolism

Under normal circumstances, cellular iron levels are maintained in balance through an iron transport system. Alterations in iron intake or export can heighten the sensitivity of tumor cells to oxidative damage and ferroptosis [92]. Ferrous iron (Fe^2+^), a key inducer of lipid peroxidation and ferroptosis, can have its levels elevated through transferrin (TF)‐mediated iron uptake or the autophagy and lysosomal degradation of ferritin. Nuclear receptor coactivator 4 (NCOA4) is a ferritin‐specific receptor that mediates the transport of ferritin to autophagosomes for ferritinophagy, and the knockdown of NCOA4 has been shown to reduce the sensitivity of human fibrosarcoma and pancreatic cancer cells to ferroptosis [93]. Iron‐responsive element‐binding protein 2 (IREB2) is a principal regulator of iron metabolism, and miR‐19a suppresses ferroptosis of colorectal cancer cells by targeting IREB2 [94]. Moreover, studies have identified the Ras‐RAF‐MEK pathway as playing a decisive role in the sensitivity of some tumor cell lines to ferroptosis. Oncogenic Ras has been observed to increase cellular iron content by downregulating the membrane iron transporter (ferroportin, FPN) and upregulating the transferrin receptor in many tumor cell lines. Due to their rapid proliferation needs, tumor cells have a higher demand for iron compared to non‐tumor cells, making them more susceptible to iron overload and ROS accumulation. This iron dependency positions iron ion‐mediated targeting in the tumor microenvironment as a viable therapeutic strategy.

### 5.3 Lipid Metabolism

Lipid peroxides exert toxic effects on tumor cells through two mechanisms. At the molecular level, lipid peroxides further decompose, consuming nucleic acids and proteins, thereby driving ferroptosis. Structurally, extensive lipid peroxidation leads to the thinning and increased curvature of biological membranes, exacerbating oxidation and ultimately causing membrane instability and micelle formation [95]. In addition, the Fenton reaction elucidates how some ferroptosis inducers exert their lethal effect primarily through iron‐containing enzymes (arachidonate lipoxygenases, ALOX) mediated lipid peroxidation. Small scaffold protein Raf1 kinase inhibitor protein positively regulates ferroptosis by binding to ALOX15 and interfering with the production of phosphatidylethanolamine. The sources of ROS involved in ferroptosis are extensive, and the accumulation of its oxidation products (especially lipid peroxides) is considered a hallmark of ferroptosis. Most tumor cells exhibit elevated ROS levels, and elevating ROS to cytotoxic levels may serve to eliminate tumor cells [96]. When ROS levels are increased, endogenous cysteine levels are insufficient for synthesizing enough GSH. Thus, external cysteine needs to be obtained through system Xc, where erastin, sorafenib, and sulfasalazine can act as inhibitors of system Xc to induce ferroptosis in tumor cells.

## 6 Conclusion

In summary, ferroptosis is a newly discovered form of cell death, with iron metabolism, lipid metabolism, and glutathione metabolism being key mechanisms in its regulation. Tumor‐infiltrating lymphocytes (TILs) are the most crucial components of the tumor immune microenvironment, determining the body's anti‐tumor immune activity and regulating the responsiveness to tumor immune therapy. This review primarily summarizes various common TILs, their sensitivity to ferroptosis, and key genes regulating their sensitivity. TAMs' ferroptosis reduced M2‐macrophage infiltration and increased polarization to M1 macrophages to promote CD8+ T cell activity. MDSCs' ferroptosis reduced the inhibition of CD8+ T cells. PMN‐MDSCs and DCs blocked the CD8+ T cell activation pathway. CD8+ T cell‐derived IFN‐γ binds to IFNγR on the surface of cancer cells and promotes tumor cell ferroptosis. Inhibition of SLC7A11/SLCA2 expression to block the uptake of cystine and reduce the activity of GPX4, which also promotes cell ferroptosis. The release of DAMPs during cancer cell ferroptosis induces the activation of CD8+ T cells, establishing a positive recycling pathway. CD36 promoted uptake of oxLDL into T cells, and this induced lipid peroxidation and downstream activation of p38 kinase. These promote T cell functional exhaustion and underscore the therapeutic potential of blocking CD36 to boost anti‐tumor immunity. In conclusion, the complex interplay between ferroptosis and the tumor microenvironment plays a critical role in modulating the effectiveness of immunotherapy. Therapeutic approaches that promote ferroptosis in tumor cells and immunosuppressive cells (such as M2‐type macrophages, Treg cells, and MDSCs), as well as reducing CD8+ T cell ferroptosis, are effective therapeutic strategies that can decrease the occurrence of resistance to immune checkpoint inhibitors. Furthermore, the integration of immunotherapeutic agents with drugs targeting ferroptosis signaling can synergistically enhance the effectiveness of immune therapy and holds promise for achieving efficient and low‐toxicity anti‐tumor effects through nanomedicine delivery systems.

## Author Contributions

**Fenfen Zhan:** data curation, methodology, writing – original draft. **Yanyan Hu:** conceptualization. **Xiang Jiang:** conceptualization. **Zejun Fang:** conceptualization, funding acquisition, investigation, writing – review and editing.

## Ethics Statement

The authors have nothing to report.

## Consent

The authors have nothing to report.

## Conflicts of Interest

The authors declare no conflicts of interest.

## Data Availability

The datasets that were utilized or analyzed in this study can be obtained from the corresponding author upon a reasonable request.
